# Outcomes of Patients With Hypothyroidism and COVID-19: A Retrospective Cohort Study

**DOI:** 10.3389/fendo.2020.00565

**Published:** 2020-08-18

**Authors:** Maaike van Gerwen, Mathilda Alsen, Christine Little, Joshua Barlow, Leonard Naymagon, Douglas Tremblay, Catherine F. Sinclair, Eric Genden

**Affiliations:** ^1^Department of Otolaryngology- Head and Neck Surgery, Icahn School of Medicine at Mount Sinai, New York, NY, United States; ^2^Institute for Translational Epidemiology, Icahn School of Medicine at Mount Sinai, New York, NY, United States; ^3^Division of Hematology and Medical Oncology, Icahn School of Medicine at Mount Sinai, New York, NY, United States

**Keywords:** COVID-19, hypothyroidism, survival, outcomes, epidemiology, cohort

## Abstract

Coronavirus diseases (COVID-19) is associated with high rates of morbidity and mortality and worse outcomes have been reported for various morbidities. The impact of pre-existing hypothyroidism on COVID-19 outcomes remains unknown. The aim of the present study was to identify a possible association between hypothyroidism and outcomes related to COVID-19 including hospitalization, need for mechanical ventilation, and all-cause mortality. All patients with a laboratory confirmed COVID-19 diagnosis in March 2020 in a large New York City health system were reviewed. Of the 3703 COVID-19 positive patients included in present study, 251 patients (6.8%) had pre-existing hypothyroidism and received thyroid hormone therapy. Hypothyroidism was not associated with increased risk of hospitalization [Adjusted Odds Ratio (OR_adj_): 1.23 (95% Confidence Interval (CI): 0.88- 1.70)], mechanical ventilation [OR_adj_: 1.17 (95% CI: 0.81–1.69)] nor death [OR_adj_: 1.07 (95% CI: 0.75–1.54)]. This study provides insight into the role of hypothyroidism on the outcomes of COVID-19 positive patients, indicating that no additional precautions or consultations are needed. However, future research into the potential complications of COVID-19 on the thyroid gland and function is warranted.

## Introduction

Coronavirus disease (COVID-19), the disease caused by severe acute coronavirus 2 (SARS-CoV-2), has spread dramatically worldwide and is associated with high rates of morbidity and mortality (1, 2). The clinical presentation ranges from an asymptomatic infection to severe viral pneumonia with acute respiratory failure, sepsis and death (3). Older age, male gender and the presence of multiple comorbidities have been identified as the main risk factors for more severe disease and worse outcomes (3, 4). There is some epidemiological evidence that certain comorbidities are associated with worse outcomes. However, the impact of hypothyroidism on outcome in COVID-19 positive patients remains unknown.

It has been established that angiotensin-converting enzyme-2 (ACE2) is the functional host receptor for SARS-CoV-2, ACE2 is expressed in various cells in different organs, including the thyroid gland (5, 6). Data on thyroid function or thyroid pathology in COVID-19 patients is not yet available although destruction of follicular cells was seen in an autopsy study of SARS infected patients published in 2007 (7). Currently, the American Thyroid Association (ATA) does not have specific recommendations for patients with hypothyroidism but underlines the importance of minimizing the spread of COVID-19 and advises patients with underlying hypothyroidism to continue taking their medication as prescribed (8).

The aims of this study are therefore to investigate the association between pre-existing hypothyroidism and COVID-19 related outcomes, including hospitalization, need for mechanical ventilation and all-cause mortality.

## Materials and Methods

### Study Population

All patients with a positive result on a reverse-transcriptase-polymerase-chain-reaction (RT-PCR) SARS-CoV-2 assay of a nasopharyngeal swab specimen and therefore diagnosed with laboratory confirmed COVID-19 between March 1, 2020 and April 1, 2020 were identified via the electronic medical record of a large New York City health system (*n* = 4,343). Both hospitalized and ambulatory patients were included. Patients were excluded if they were <18 years old (*n* = 55) or had insufficient clinical documentation available or accessible, including confidential patient records (*n* = 585), resulting in a final study population of 3703 COVID-19 positive patients. This study was approved by the Program for the Protection of Human Subjects (PPHS) of the Icahn School of Medicine at Mount Sinai.

### Data Collection

The medical records of all patients were retrospectively reviewed and data relevant to our study was collected and securely stored using Research Electronic Data Capture software (REDCap, Vanderbilt University). COVID-19 patients were identified as having hypothyroidism as a comorbidity when (1) the significant medical history in the medical record mentioned “hypothyroidism” (ICD-9 code 244, 245.2 or ICD-10 code E02, E03, E06.3) or (2) the term hypothyroidism was found within the clinical notes, combined with receiving thyroid hormone therapy before the COVID-19 related hospital visit/ admission. Data was collected on age, sex, race, smoking status, and body mass index (BMI) with the cut-offs used for normal weight (<25 kg/m^2^), overweight (25–30 kg/m^2^) and obese (>30 kg/m^2^), as proposed by the Center for Disease Control and Prevention (CDC) (9). Data on comorbidities was collected and translated into a categorical variable on the number of comorbidities. Survival time was calculated as the time (days) between a positive result on a RT-PCR SARS-CoV-2 assay of a nasopharyngeal swab specimen and last follow-up. Our primary predictor of interest was the presence of hypothyroidism. Data on the primary outcomes of interest was collected up to May 13, 2020 and included hospital admission, need for invasive mechanical ventilation (i.e., intubation), and all-cause mortality.

### Statistical Analysis

Demographic and clinical characteristics were compared between the hypothyroidism group and the no hypothyroidism group using two-sided *t*-test for age and chi^2^ tests for the categorical variables. Adjusted analysis was performed using multivariable logistic regression to calculate the odds of hospitalization between the hypothyroidism and the no hypothyroidism group, adjusting for age, sex, race, BMI, smoking status and number of comorbidities. Multivariable logistic regression was also used to calculate the odds of mechanical ventilation and death between the hypothyroidism and the no hypothyroidism group within the group of hospitalized patients.

We additionally applied propensity score matching methods to deal with potential bias due to non-random treatment allocation among COVID-19 positive patients with and without hypothyroidism (10). Propensity scores were calculated using a logistic regression model adjusting for age, sex, race, BMI, smoking status, and number of comorbidities. A one-to-three matching technique was utilized by matching one patient from the hypothyroidism group to three patients from the no hypothyroidism group based on their propensity score values, using the Greedy matching technique (10). Event analysis for the outcome hospitalization was determined. Within the group of hospitalized patients, we again matched one patient from the hypothyroidism group to three patients from the no hypothyroidism group based on their propensity score values and ran event analysis for the outcome mechanical ventilation and all-cause mortality. We additionally performed time-to-event analysis for all-cause mortality. Patients with a survival time exceeding 50 days were censored to avoid a small number of patients at risk. The results of time-to-event analysis were expressed as a Kaplan-Meier curve with significance indicated using log-rank test. Cox proportional hazard model of all-cause mortality with robust sandwich variance estimates of standard errors was performed and expressed as hazard ratio. All statistical analyses were performed using SAS 9.4 (SAS Institute Inc., Cary, NC).

## Results

Our study population consisted of 3703 COVID-19 positive patients, of which 251 patients (6.8%) had pre-existing hypothyroidism. Of the 251 patients with pre-existing hypothyroidism, 22 patients (8.8%) had Hashimoto's disease. Patients in the hypothyroidism group were significantly older, more frequently female and Non-Hispanic White and had significantly more other comorbidities (Table 1); 68.1% of the COVID-19 positive patients with hypothyroidism needed hospitalization.

**Table 1:** Demographic and clinical characteristics of the study population by hypothyroidism status (*n* = 3,703).

	**Hypothyroidism (*n* = 251) *n* (%)**	**Without hypothyroidism (*n* = 3,452) *n* (%)**	***p*-value**
Age (years ± SD)	65.0 (± 16.9)	56.4 (±18.2)	<0.001
Female	173 (68.9)	1,481 (42.9)	<0.001
Race			<0.001
NHW	114 (45.4)	899 (26.0)	
NHB	39 (15.5)	953 (27.6)	
Other/ unknown	98 (39.0)	1,600 (46.4)	
Smoking			<0.001
Never	153 (61)	1,991 (57.7)	
Former	72 (28.7)	641 (18.6)	
Current	7 (2.8)	186 (5.4)	
Unknown	19 (7.6)	634 (18.4)	
BMI			<0.001
<25	74 (29.5)	771 (22.3)	
25-30	75 (29.9)	981 (28.4)	
> 30	87 (34.7)	985 (28.5)	
Unknown	15 (6.0)	715 (20.7)	
Number of Comorbidities^*^			<0.001
0	55 (21.9)	1,305 (37.8)	
1	52 (20.7)	786 (22.8)	
2	40 (15.9)	575 (16.7)	
>2	104 (41.4)	786 (22.8)	
Hospital admission	171 (68.1)	1,844 (53.4)	<0.001

*NHB, Non-Hispanic Black; NHW, Non-Hispanic White; SD, standard deviation*.

* *Comorbidities include hypertension, coronary artery disease, atrial fibrillation, congestive heart failure, peripheral vascular disease, cerebrovascular accident/ transient ischemic attack, dementia, diabetes, chronic kidney disease stage III or greater, malignancy (including all types of cancer as well as lymphoma and leukemia), asthma, chronic obstructive pulmonary disease and prior venous thromboembolism*.

### Hospitalization

Hypothyroidism was not associated with increased risk of hospitalization [Adjusted Odds Ratio (OR_adj_): 1.23 (95% Confidence Interval (CI): 0.88–1.70)] (Table 2). Propensity score matching yielded 241 patients with hypothyroidism and 723 patients without hypothyroidism with balanced variables between the groups (Supplementary Table 1). The risk of hospitalization was not statistically significantly different between the matched groups [OR: 0.76 (95% CI: 0.58–1.00)] (Table 2).

**Table 2:** Association between hypothyroidism and COVID-19 outcomes.

	Adjusted analysis^*^ OR_**adj**_ (95% CI)	**Propensity matched analysis OR (95% CI)**
Hospitalization	1.23 (0.88–1.70)	0.76 (0.58–1.00)
Mechanical ventilation^#^	1.17 (0.81–1.69)	0.85 (0.58–1.25)
Death^#^	1.07 (0.75–1.54)	1.04 (0.71–1.52)

* *Adjusted for age, sex, race, BMI, smoking status, number of comorbidities*.

# *Only included hospitalized patients*.

### Mechanical Ventilation and Mortality in Hospitalized Patients

**Figure 1:**
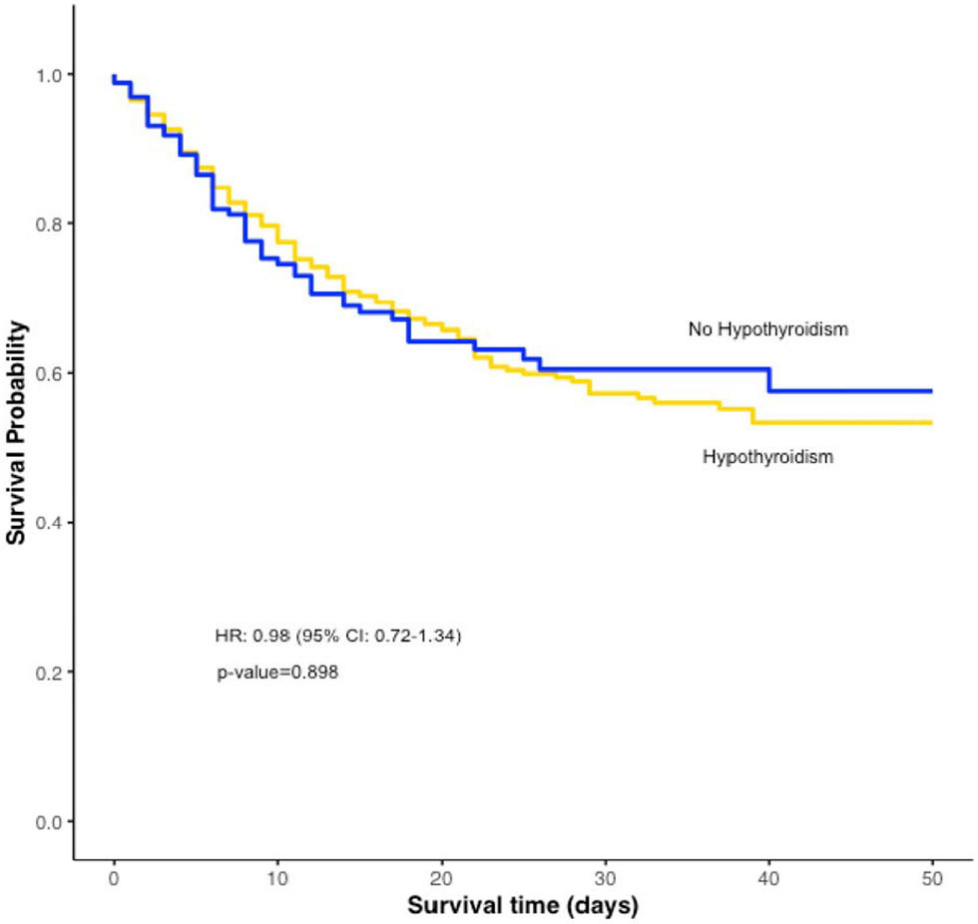
Overall survival in the propensity matched hypothyroidism- no hypothyroidism cohort.

In the group of hospitalized patients (*n* = 2,015), hypothyroidism was not associated with an increased risk of mechanical ventilation [OR_adj_: 1.17 (95% CI: 0.81–1.69)] or death [OR_adj_: 1.07 (95% CI: 0.75–1.54)] (Table 2). Propensity score matching yielded 158 hospitalized patients with hypothyroidism and 474 hospitalized patients without hypothyroidism with balanced variables between the groups (Supplementary Table 2). Hypothyroidism was not associated with increased risk of mechanical ventilation [OR: 0.85 (95% CI: 0.58–1.25)] or death [OR: 1.04 (95% CI: 0.71–1.52)] (Table 2). There was no statistically significant difference in survival (p= 0.898) between the two groups [HR: 0.98 (95% CI: 0.72–1.34)] (Figure 1).

## Discussion

This large, retrospective cohort study showed that hypothyroidism is not associated with increased risk of COVID-19 related hospitalization or a worse outcome, including death. The current recommendations by the ATA for patients with hypothyroidism are therefore accurate and no additional precautions are needed for patients suffering from hypothyroidism.

From published literature, we know that well-managed hypothyroidism is not associated with increased infection risk although there is some evidence that susceptibility to infection might increase in patients with poorly controlled hypothyroidism (11, 12). Therefore, the treatment of thyroid disorders in COVID-19 positive patients remains of significant importance amid the ongoing pandemic. The unprecedented strain on medical resources and implementation of social distancing measures pose a fundamental challenge to the treatment of patients with chronic conditions, including hypothyroidism. Hypothyroidism requires management with thyroid hormone replacement therapy and active monitoring through regular biochemical testing to ensure that therapeutic levels are maintained (13). Iatrogenic overtreatment with thyroid hormone supplementation is a known risk factor for the development of thyrotoxicosis (14). A joint statement by the British Thyroid Association and the Society for Endocrinology (BTA/SfE) as well as the ATA regarding the COVID-19 pandemic therefore strongly advise that patients with thyroid disease continue taking their thyroid medications as prescribed in order to reduce any risk of thyroid dysregulation (15) that could lead to a more severe COVID-19 outcome.

Besides pre-existing hypothyroidism as potential focus of interest, there is some evidence that endocrinological disruption and destruction of thyroid tissue may be a complication of COVID-19, even in patients without pre-existing endocrinological conditions (7, 16–19). It is known that SARS-CoV, the virus responsible for the SARS outbreak in 2003, as well as SARS-CoV-2 use ACE2 to enter human cells (20, 21). ACE2 is expressed by various cells in the body, including the thyroid gland (5, 6). Destruction of thyroid gland tissue and temporary or permanent thyroid dysfunction should therefore be subject of future studies.

This study was a retrospective study using data collected from the electronic medical records of ambulatory and hospitalized COVID-19 positive patients. A limitation of this study includes that the study population consisted solely of patients within the New York metropolitan area therefore potentially limiting the generalizability of the results. Although assessing the outcomes of patients with hypothyroidism due to Hashimoto's disease compared to patients with hypothyroidism due to other causes would be clinically relevant, a subgroup analysis was not feasible because of too low number of patients with Hashimoto's disease in our study cohort. The increased patient volume and reduced consultation time per patient associated with the increased influx of patients during the pandemic potentially resulted in missing data, especially for ambulatory patients, on certain covariates including race, BMI, and smoking status. Furthermore, we were unable to collect mortality data for the patients who were still hospitalized at the time of data collection, therefore potentially introducing bias. However, we estimate that little bias was introduced because only 1.3% of the study population was still hospitalized at the time of data collection.

To our knowledge, this is the first study investigating the COVID-19 associated outcomes in patients with pre-existing hypothyroidism in a population drawn from the first and largest COVID-19 epicenter in the US, with sufficiently long follow-up to rigorously assess whether pre-existing hypothyroidism is a risk factor for worse outcomes.

In conclusion, hypothyroidism is not a risk factor associated with worse outcomes in COVID-19 positive patients, therefore no additional precautions or consultations are needed. However, future research into the potential complications of COVID-19 on the thyroid gland and function is warranted.

## Data Availability Statement

The raw data supporting the conclusions of this article will be made available by the authors, without undue reservation.

## Ethics Statement

This study was approved by the Program for the Protection of Human Subjects (PPHS) of the Icahn School of Medicine at Mount Sinai.

## Author Contributions

All authors have made substantial contributions to the conception or design of the work (CS, MG, MA, CL, DT, EG), or the acquisition, analysis, or interpretation of data, or the creation of new software used in the work (MG, MA, CL, JB, LN, DT, CS, EG), or have drafted the work or substantively revised it (MG, MA, CL, CS), and have approved the submitted version (plus any substantially modified version that involves the author's contribution to the study [all authors]), and to have agreed both to be personally accountable for the author's own contributions and to ensure that questions related to the accuracy or integrity of any part of the work, even ones in which the author was not personally involved, are appropriately investigated, resolved, and the resolution documented in the literature.

## Conflict of Interest

The authors declare that the research was conducted in the absence of any commercial or financial relationships that could be construed as a potential conflict of interest. The handling Editor declared a past co-authorship with one of the author CS.
