# Supplementary material for: Outcomes of Patients With Hypothyroidism and COVID-19: A Retrospective Cohort Study
Source: Front Endocrinol (Lausanne). 2020 Aug 18;11:565. doi: 10.3389/fendo.2020.00565 (PMC7461836; doi:10.3389/fendo.2020.00565)
Supplement: Supplementary file 1 [file Table_1.DOCX]

**Supplementary table 1:** Demographic and clinical characteristics of the propensity matched hypothyroidism /- no hypothyroidism population for all patients

|  | **Hypothyroidism (n=241)**  **n (%)** | **Without hypothyroidism (n=723)**  **n (%)** | **p-value** |
| --- | --- | --- | --- |
| **Age** (years ± SD) | 64.3 (± 16.8) | 64.7 (±17.3) | 0.698 |
| **Male** | 78 (32.4) | 236 (32.6) | 0.937 |
| **Race** |  |  | 0.951 |
| NHW | 104 (43.2) | 308 (42.6) |  |
| NWB | 39 (16.2) | 113 (15.6) |  |
| Other/ unknown | 98 (40.7) | 302 (41.8) |  |
| **Smoking** |  |  | 0.95 |
| Never | 148 (61.4) | 434 (60.0) |  |
| Former | 67 (27.8) | 213 (29.5) |  |
| Current | 7 (2.9) | 18 (2.5) |  |
| Unknown | 19 (7.9) | 58 (8.0) |  |
| **BMI** |  |  | 0.954 |
| <25 | 70 (29.1) | 203 (28.1) |  |
| 25-30 | 72 (29.9) | 229 (31.7) |  |
| > 30 | 84 (34.8) | 244 (33.7) |  |
| Unknown | 15 (6.2) | 47 (6.5) |  |
| **Number of Comorbidities** |  |  | 0.802 |
| 0 | 55 (22.8) | 156 (21.6) |  |
| 1 | 50 (20.7) | 156 (21.6) |  |
| 2 | 40 (16.7) | 138 (19.1) |  |
| >2 | 96 (39.8) | 273 (37.7) |  |

NHB= Non-Hispanic Black

NHW= Non-Hispanic White

SD= standard deviation

*Comorbidities include hypertension, coronary artery disease, atrial fibrillation, congestive heart failure, peripheral vascular disease, cerebrovascular accident/ transient ischemic attack, dementia, diabetes, chronic kidney disease stage III or greater, malignancy (including all types of cancer as well as lymphoma and leukemia), asthma, chronic obstructive pulmonary disease and prior venous thromboembolism
